# Tryptophan Metabolism via Kynurenine Pathway: Role in Solid Organ Transplantation

**DOI:** 10.3390/ijms22041921

**Published:** 2021-02-15

**Authors:** Ruta Zulpaite, Povilas Miknevicius, Bettina Leber, Kestutis Strupas, Philipp Stiegler, Peter Schemmer

**Affiliations:** 1 General, Visceral and Transplant Surgery, Department of Surgery, Medical University of Graz, Auenbruggerpl. 2, 8036 Graz, Austria; ruta.zulp@gmail.com (R.Z.); povilui.m@gmail.com (P.M.); bettina.leber@medunigraz.at (B.L.); peter.schemmer@medunigraz.at (P.S.); 2 Faculty of Medicine, Vilnius University, M. K. Ciurlionio 21, 03101 Vilnius, Lithuania; kestutis.strupas@santa.lt

**Keywords:** transplantation, ischemia-reperfusion, tolerance, rejection, tryptophan, kynurenine, indoleamine-2,3-dioxygenase

## Abstract

Solid organ transplantation is a gold standard treatment for patients suffering from an end-stage organ disease. Patient and graft survival have vastly improved during the last couple of decades; however, the field of transplantation still encounters several unique challenges, such as a shortage of transplantable organs and increasing pool of extended criteria donor (ECD) organs, which are extremely prone to ischemia-reperfusion injury (IRI), risk of graft rejection and challenges in immune regulation. Moreover, accurate and specific biomarkers, which can timely predict allograft dysfunction and/or rejection, are lacking. The essential amino acid tryptophan and, especially, its metabolites via the kynurenine pathway has been widely studied as a contributor and a therapeutic target in various diseases, such as neuropsychiatric, autoimmune disorders, allergies, infections and malignancies. The tryptophan-kynurenine pathway has also gained interest in solid organ transplantation and a variety of experimental studies investigating its role both in IRI and immune regulation after allograft implantation was first published. In this review, the current evidence regarding the role of tryptophan and its metabolites in solid organ transplantation is presented, giving insights into molecular mechanisms and into therapeutic and diagnostic/prognostic possibilities.

## 1. Introduction

Solid organ transplantation (Tx) remains the gold standard and the only curative treatment for patients with end-stage organ disease. A substantial improvement in patient and graft survival has been observed during the last decades, mainly due to technical developments and advancements in immunosuppressive regimens [1]. However, there are not sufficient amounts of donor organs to treat all patients on the waiting list for whole organ Tx [2,3,4]. Therefore, it is inevitable to utilize organs retrieved from extended criteria donors (ECD). These grafts are known to be extremely susceptible to ischemia-reperfusion injury (IRI), often resulting in delayed graft function (DGF) or primary non-function (PNF) after implantation. Therefore, additional efforts to diminish the harm inflicted during organ preservation techniques are necessary [5,6].

The problem of allograft rejection is another important challenge limiting Tx success. The increased variety of effective immunosuppressants resulted in a substantial reduction of acute graft loss rates [7,8]. However, overall graft survival still remains time-limited, mainly due to chronic rejection and subsequent chronic allograft dysfunction [7,8]. The increased risk of organ rejection especially poses a barrier for highly sensitized patients who were already exposed to an antigen or need a repeated Tx [9]. 

Moreover, specific and sensitive biomarkers to predict organ quality and Tx success are needed in order to expand the potential donor organ pool and transplant the organs safely. Biomarkers for the detection and diagnosis of rejection and/or allograft dysfunction, which would allow a more thorough follow-up and personalized care for recipients at risk, is also of particular interest [10,11] to ameliorate the long-term results after Tx.

Tryptophan is one of the eight essential amino acids not synthesized in the human body and, hence, needs to be supplemented with the nutriment. Among all essential amino acids, the tryptophan concentration is the lowest in the human organism; however, only low concentrations are sufficient for tryptophan-involved processes [12]. Actually, less than 1% of the available tryptophan is used for protein synthesis, while the remaining 99% serves as a precursor of bioactive metabolites, including serotonin, tryptamine, melatonin and kynurenine, as well as the essential coenzyme nicotinamide adenine dinucleotide (NAD+) [13,14]. The role of tryptophan has been extensively studied in a variety of conditions, such as neuropsychiatric disorders [15]; autoimmune and allergic diseases [16,17]; infections [17]; brain tumors [18] and other cancer types, e.g., breast, bladder, colorectal cancers and melanomas [17,19,20]. Tryptophan metabolism via the kynurenine pathway, which is the main route of tryptophan degradation in the human body [14,21,22], has gained particular interest in Tx during the last couple of decades. Since the landmark discovery by Munn et al., who showed that the rejection of an allogeneic fetus in mice is prevented by indoleamine-2,3-dioxygenase (IDO)-mediated tryptophan catabolism [23], enzymes and metabolites of the tryptophan-kynurenine pathway have been widely studied in the field of immune regulation after Tx [17]. In addition, tryptophan and its metabolites also seem to play a role in IRI, thus giving some new direction in the field of organ preservation [24,25]. Most interestingly, inflammation is the basic underlying phenomenon for graft pathology, including IRI and both acute and chronic graft rejections [7,26].

This comprehensive review gives an overview on the current evidence of the tryptophan-kynurenine pathway’s significance in Tx, giving both diagnostic/prognostic and therapeutic implications.

## 2. The Tryptophan-Kynurenine Pathway, Its Enzymes and Metabolites: An Overview

**Figure 1:**
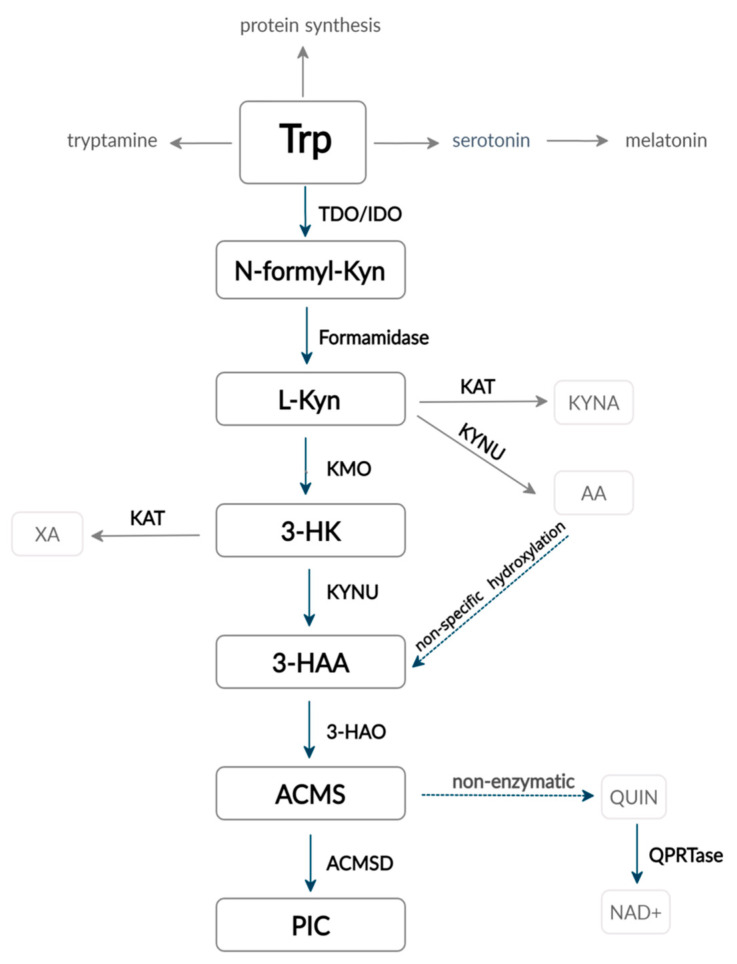
Tryptophan metabolism via the kynurenine pathway. Abbreviations: Trp: tryptophan, N-formyl-Kyn: N-formyl-kynurenine, Kyn: kynurenine, 3-HK: 3-hydroxykynurenine, 3-HAA: 3-hydroxyanthranilic acid, ACMS: 2-amino-3-carboxymuconate-semialdehyde, PIC: picolinic acid, KYNA: kynurenic acid, AA: anthranilic acid, XA: xanturenic acid, QUIN: quinolinic acid, NAD+: nicotinamide adenine dinucleotide, TDO: tryptophan-2,3-dioxygenase, IDO: indoleamine-2,3-dioxygenase, KMO: kynurenine-3-monooxygenase, KYNU: kynureninase, 3-HAO: 3-hydroxyanthranilate-3,4-dioxygenase, ACMSD: amino-carboxy-muconate-semialdehyde-decarboxylase and KAT: kynurenine aminotransferase.

There is evidence that ~90–95% of the overall tryptophan is degraded via the kynurenine pathway, and it is the main route of tryptophan catabolism in the human body [21,22] (Figure 1).

Firstly, tryptophan is converted to N-formyl-L-kynurenine. The reaction is catalyzed by one of three rate-limiting enzymes: tryptophan-2,3-dioxygenase (TDO) and indoleamine-2,3-dioxygenase 1 or 2 (IDO1 or IDO2). All the three enzymes are hemoproteins and use molecular O_2_ as a co-substrate, which also allows them to utilize reactive oxygen species (ROS) and regulate the redox balance in the cell [17,27,28]. Unless TDO has been detected in various mouse brain structures, as well as in different tumor types [29,30], this enzyme is mainly expressed in the liver and regulates systemic tryptophan levels in physiological conditions [20,31]. Glucocorticoids, tryptophan itself and heme are the three main regulators of TDO: glucocorticoids induce the synthesis of new TDO apoenzyme, while the substrate tryptophan enhances the conjugation of the apoenzyme with the cofactor heme and stabilizes the whole structure. TDO transcription has also been found to be upregulated by glucagon but inhibited by insulin and adrenaline [32]. Other possible inhibitors described in the literature include reduced forms of nicotinamide adenine dinucleotide phosphate (NAD(P)H), 3-hydroxykynurenine (3-HK) and 3-hydroxyanthranilic acid (3-HAA), as they likely act through the negative feedback mechanism; however, the results obtained by in vitro and in vivo experiments are contradictory [31].

Differently to TDO, IDO is much more widely distributed among tissues and mainly expressed by various immune cells. Interestingly, IDO has a lower capacity but much higher affinity for tryptophan than TDO [33]. Two forms of IDO are currently known: IDO 1 (previously called IDO) and the more recently discovered IDO 2, which is expressed in human liver, kidney and brain [17]. Tryptophan and the cofactor heme both increase IDO activity in a similar way as TDO; however, it was found that high concentrations of tryptophan act as inhibitor of IDO [20,34,35]. Antioxidants [36,37], as well as nitric oxide (NO), are also known to inhibit IDO function [38]. Nevertheless, the principal effectors of IDO activity are pro- and anti-inflammatory cytokines and mediators [17], which will be discussed in more detail below.

In the next step, N-formyl-kynurenine form amidase hydrolyzes N-formyl-L-kynurenine to L-kynurenine, which is subsequently transformed into three alternative metabolites with different properties regarding oxidative stress and organ toxicity: (1) kynurenic acid (KYNA) by kynurenine aminotransferase (KAT), (2) anthranilic acid (AA) by kynureninase (KYNU) and (3) 3-hydroxykynurenine (3-HK) by kynurenine-3-monooxygenase (KMO). The latter metabolite 3-HK can be further converted into xanthurenic acid (XA) by KAT or transformed into 3-hydroxyanthranilic acid (3-HAA) by KYNU [17,20].

The next and the most potent enzyme in the tryptophan-kynurenine pathway is 3-hydroxyanthranilate-3,4-dioxygenase (3-HAO), which rapidly converts 3-HAA into the unstable 2-amino-3-carboxymuconate-semialdehyde (ACMS). The latter is then nonenzymatically converted into quinolinic acid (QUIN) used in NAD+ and NADP+ formation or, alternatively, transformed into picolinic acid (PIC) via an amino-carboxy-muconate-semialdehyde-decarboxylase (ACMS) catalyzed reaction [17,20]. 3-HAA and 3-HK are known generators of free radicals [39]; however, there is evidence that the same metabolites are also able to act as antioxidants, depending on the cell’s redox properties [40]. Similarly, PIC and QUIN also enhance oxidative stress by creating free radicals. On the other hand, KYN catalyzation to KYNA by KATs is considered an antioxidant way of tryptophan metabolism. As a result, KMO is located at an important point keeping the balance between anti- and pro-oxidant metabolites [20]. It is important to mention that, due to the high K*m* (concentration of substrate that permits the enzyme to achieve half of the maximum velocity of its catalyzed reaction) of KAT for its substrates, KAT reactions are only minimally significant, whereas the KMO/KYNU catalyzed branch remains the main one. Nevertheless, KAT can be enhanced by the overload of tryptophan or kynurenine or by the inhibition of KMO activity [41].

Importantly, under physiologic conditions, the tryptophan-kynurenine pathway happens mainly in the liver, where all the enzymes necessary for NAD+ synthesis from tryptophan are present. The extrahepatic way is responsible for only 5–10% of the overall tryptophan degradation in physiologic conditions. However, it becomes much more significant under immunological circumstances, inflammation and oxidative stress. The extrahepatic tryptophan kynurenine pathway does not provide all the necessary enzymes; therefore, its intermediate metabolites and their properties become critical in the pathogenesis and modulation of these conditions [17,20].

## 3. Role of Tryptophan and Its Metabolites in IRI

IRI is a substantial and unavoidable threat in Tx, determining the early transplant function. Adenosine triphosphate (ATP) depletion, impaired ATPases activity, cellular calcium overload, deterioration of the mitochondrial membrane potential via opened mitochondrial permeability transition pores, the promotion of proapoptotic mechanisms, the generation of ROS after the reintroduction of molecular oxygen, endothelial dysfunction, increased thrombogenicity and the induction of inflammatory responses are examples for the consequences of IRI [26]. Unless the role of tryptophan and its metabolism in IRI has been studied to a much lesser extent compared to immune responses after Tx, several implications have been given in the literature that could lead to further investigations (Table 1).

The histidine-tryptophan-ketoglutarate (HTK) organ preservation solution, which is nowadays considered one of the standard solutions used in Tx, contains 2-mmol/L tryptophan due to its antioxidant capacity and membrane stabilizing potential [42]. On the contrary, several studies have suggested that tryptophan starvation could be a protective factor against IRI. It has been previously demonstrated that short-term dietary restrictions, i.e., reductions of specific food intakes without calorie depletion, increases the resistance to acute stress, including IRI [43,44]. In another experimental study, the total protein or single amino acid L-tryptophan withdrawal from diet protected mice against renal ischemic injury and preserved kidney function. In addition, tryptophan starvation resulted in significantly reduced circulating granulocyte numbers, which was found to be general control non-depressible 2 (GCN2) kinase-dependent [45]. Nevertheless, the same experiments showed that tryptophan withdrawal downregulates the expression of oxidative stress resistance-related genes glutathione synthetase (Gst1/2), catalase, dehydrogenase quinone 1 (Nqo1) and NADPH in the liver [45]. Recently, Eleftheriadis et al. demonstrated a three-fold increase in human renal proximal tubular epithelial cell survival under hypoxic conditions following tryptophan deprivation. They found these effects to be driven by induction of autophagy through the activation of GCN2 kinase and p53-mediated BNIP3L upregulation [46]. Tryptophan deficiency stimulates GCN2 kinase by promoting phosphorylation of the eukaryotic translation initiation factor 2a (eIF2a), which then activates autophagy, while tryptophan supplementation acts the opposite by diminishing activity in the GCN2-eIF2a pathway [47]. In general, autophagy was confirmed to be protective in the situation of acute kidney injury caused by ischemia and reperfusion [48,49]. During the process of autophagy, cytoplasmic fractions are sequestrated within the autophagosomes, which then fuse with lysosomes, where the “captured” material is decomposed. Autophagy is activated in response to various stressors, such as hypoxia or nutrient deprivation, thereby counteracting apoptosis and providing necessary nourishment and time for cells to adapt and survive [46,47,48,49]. Moreover, tryptophan starvation-induced autophagy was found to dampen the secretion of proinflammatory cytokines [47]. These interesting results allow speculating that donor kidney preconditioning by tryptophan deprivation could induce the resistance of kidney tissue to hypoxic stress, prevent acute kidney injury and probably decrease the risk of primary nonfunction or delayed graft function after implantation.

IDO has also been investigated concerning its impact in the IRI setting. Transplanted lungs are, compared to other organs, especially prone to IRI due to the vast and constant exposition to environmental oxygen. Reactive oxygen species cause endothelial dysfunction and increase the vascular permeability and extravasation of inflammatory cells, which promotes apoptosis and necrosis processes and causes pulmonary graft injury [50]. As mentioned before, IDO, while catalyzing the reaction of L-tryptophan to L-kynurenine, consumes O_2_ and utilizes superoxide anion radicals, thus acting as a powerful antioxidant [27]. Indeed, intravenous nonviral IDO gene delivery to donor rats 24 h before lung Tx significantly protects lung allografts against IRI by reducing the vascular leak and leukocyte extravasation. Furthermore, it preserves a histological structure of the graft and significantly improves the lung function. In vitro experiments showed that enhanced IDO activity in endothelium stabilizes the intracellular redox balance and preserves the mitochondrial structure and function in the context of oxidative stress [28]. On the contrary, Mohib et al. raised concerns regarding the role of IDO in kidney graft protection, showing that, despite the pro-tolerant properties of this enzyme, its activity augments IRI in the kidney [51,52]. They found increased apoptosis in renal tubular cells due to upregulated IDO activity [51], while the inhibition of IDO by 1-metyltryptophan (1-MT) or IDO gene knockout in mice diminished renal IRI [52]. Correspondingly, another study revealed that inhibiting IDO activity by 1-MT changes the transcriptome characteristic to IRI. IRI alone was shown to change 105 coding genes and only three noncoding RNA transcripts. In mice pretreated with 1-MT, only 18 sequences of coding transcripts were altered, while the number of noncoding RNA genes was expanded to 66. The authors speculated that the reduction of IRI affects genes, including those responsible for apoptosis and cell death, and may be related to the reno-protective effects of IDO inhibition [53].

A recent study on normothermic and sub-normothermic machine perfusion for discarded human livers without significant steatosis revealed an increase in tryptophan metabolism via the kynurenine pathway and higher levels of kynurenine and KYNA for organs subjected to normothermic perfusion compared to sub-normothermic perfusion [24]. As it is known that KAT, which converts kynurenine to KYNA, is a temperature-dependent enzyme and KYNA has antioxidant properties [54], decreased KYNA production could be considered as a disadvantage of sub-normothermic perfusion. On the other hand, this finding implicates the therapeutic possibilities, e.g., shunting the kynurenine metabolism from 3-HK to KYNA by suppression of the KMO enzyme [54,55] could be beneficial in sub-normothermic perfusion conditions diminishing IRI. However, further studies using machine perfusion as a platform for tryptophan metabolism regulation and assessing the clinical outcomes of such organs are necessary.

KMO has been found to be expressed in the human kidney proximal tubule’s epithelial cells [56]. As explained above, 3-HK is an injurious metabolite of kynurenine, which enhances oxidative stress, causes pathological protein crosslinking and promotes apoptosis, while KYNA protects cells against injury in stress or inflammatory situations [17,20]. Several experimental studies revealed that the pharmacological or transcriptional blockage of KMO prevents acute ischemic kidney injury in multiorgan dysfunctional models [57,58,59,60]. Zheng et al. showed that genetically modified mice, lacking a functional KMO gene, were protected against the deleterious effects of kidney IRI. The histological necrotic tubular damage was significantly diminished, and plasma creatinine concentrations were lower compared to wild-type mice. Additionally, the number of apoptotic renal tubular cells and neutrophil infiltration into kidney tissue was significantly lower in KMO knockout mice. These findings underpin the idea of KMO as a potential therapeutic target in the prevention of IRI in the field of Tx [25]. Furthermore, a recent study, investigating glaucoma treatment possibilities, revealed that not only KMO knockout but, also, intravenous or local administration of KYNA itself acts protectively against the IRI in retinal ganglion cells [61], which could also be considered as one of the possibilities for the protection of transplanted organs.

Besides the impact of tryptophan and its metabolites on IRI of solid organ grafts, the role of tryptophan derivatives has recently been under investigation. N-acetyl-L-tryptophan (L-NAT), a ROS scavenger, inhibitor of cytochrome c release from the mitochondria, as well as an antagonist of the neurokinin 1 receptor, which has already been approved for the management of nausea, vomiting, shock and neurodegenerative diseases, was investigated in the context of hepatic IRI. The pretreatment of rats with L-NAT before liver ischemia significantly diminished IRI, prevented morphological changes of hepatocytes and increased their viability. This protective effect was found to be mediated by downregulation of the receptor interacting protein (RIP) 2/caspase1/IL-1b signaling pathway [62]. The same group later demonstrated that L-NAT protects hepatocytes against IRI by the inhibition of excessive autophagy, mitophagy and preservation of the mitochondrial structure and function [63]. Another tryptophan derivative investigated was 5-metoxytryptophan (5-MTP), an endogenous anti-inflammatory endothelial factor synthesized from L-tryptophan catalyzed by tryptophan hydroxylase-1 and hydroxyindole O-methyltransferase. An in vitro 5-MTP treatment protected cardiomyocytes against ROS-induced IRI by preventing cell death, stimulating cell migration and promoting wound healing via cytoskeletal regulations, as well as regulating the intracellular redox state and reducing the endoplasmic reticulum stress [64].

To conclude, unless the current evidence confirms the impact of tryptophan and its metabolites in graft IRI, revealing important implications in possible therapeutic approaches, which potentially would allow to diminish the burden of IRI, it seems that the effect is complex rather than one-sided. Therefore, many questions remain unanswered.

**Table 1:** Effects of tryptophan and its metabolism via the kynurenine pathway in solid organ IRI in animal models.

Reference	Experimental Model	Treatment/Intervention	Outcomes
Peng et al. [45], 2012	Mice kidney IRI model	Tryptophan deficient diet for 6 days before induction of kidney or liver ischemia.	↓serum creatinine and urea 1 day after bilateral renal ischemia-reperfusion; ↓expression of KIM-1 mRNA; ↓level of acute tubular necrosis in histology; ↓serum levels of ALT, AST, LDH after liver ischemia-reperfusion; ↓P-selectin and IL-6 gene expression; ↓number of circulating neutrophils. Effect dependent on GCN2.
Liu et al. [28], 2007	Rats model of lung Tx after 5 h of warm ischemia	Sleeping beauty transposon mediated hIDO gene delivery to donor animals intravenously 24 h prior to Tx.	↓apoptosis of endothelial cells; ↓leukocyte infiltration; ↑antioxidant capacity; ↓levels of oxidative stress markers (protein carbonyl, MDA); ↓alveolar edema, hemorrhage and formation of focal congestion of lung tissue; preserved mitochondrial structure and function; ↓peak airway pressure, ↑PaO_2_.
Mohib et al. [52], 2008	Mice kidney IRI model	IDO gene knock-out or IDO inhibition by intraperitoneal injections of 3 mg of 1-MT twice a day for 48 h following reperfusion. Some mice received 1-MT 1 h before ischemia, as well as following reperfusion.	↓serum creatinine and blood urea nitrogen in IDO-knockout mice; ↓blood urea nitrogen but no difference in serum creatinine in 1-MT-treated mice; preserved architecture of kidney tissue; ↓apoptosis and necrosis; ↓neutrophil infiltration in IDO-knockout and 1-MT treated mice.
Merchen et al. [53], 2014	Rats kidney IRI model	IDO inhibition by pretreating rats with 1-MT 140 mg/kg po 1 and 24 h prior to renal ischemia.	IRI alone changed 105 coding genes and 3 noncoding RNA transcripts. In IRI rats pretreated with 1-MT, altered coding transcripts declined to 18 sequences and altered noncoding RNA genes increased to 66.
Zheng et al. [25], 2019	Mice kidney IRI model	KMO gene knockout.	↓plasma creatinine; ↓tubular damage; ↓number of apoptotic cells; ↓neutrophil infiltration; ↓Cxcl 1 and Cxcl2 mRNA levels in kidney tissue.
Wang et al. [62], 2019 Li et al. [63], 2020	Rats liver IRI mode	Intraperitoneal administration of L-NAT (10 mg/kg) 30 min before ischemia.	↓IRI-induced histological changes in hepatocytes; ↓mRNA expression of RIP2, caspase-1 and IL-1b [62]; ↓caspase-1 activity and IL-1b expression; ↓expression of autophagy markers: LC3-II, Beclin1, and ATG-7 and ↑expression of P62; ↓formation of autophagosome; improved morphological and functional changes of mitochondria, maintained the quantity and quality of mtDNA stability; ↓excessive mitophagy [63].

Abbreviations: IRI: ischemia-reperfusion injury, Tx: transplantation, (h)IDO: (human) indoleamine-2,3-dioxygenase, KMO: kynurenine-3-monooxygenase, 1-MT: 1-metyltryptophan, L-NAT: N-acetyl-l-tryptophan, KIM: kidney injury molecule, ALT: alanyl aminotransferase, AST: aspartate aminotransferase, LDH: lactate dehydrogenase, IL: interleukin, GCN2: general control nonderepressible 2 kinase, MDA: malondialdehyde, PaO2: partial oxygen pressure, (m)RNA: (messenger) RNA, Cxcl: reduced chemokine (C-X-C motif) ligand, RIP: receptor interacting protein, LC3-II: microtubule-associated protein 1 light chain 3-II, ATG-7: autophagy-related protein-7 and mtDNA: mitochondrial DNA, ↑: increased, ↓: decreased.

## 4. The Role of the Tryptophan-Kynurenine Pathway in Immune Regulation after Tx

The immune system plays a key role in Tx. Immunological mechanisms, which normally act as a protective organism response against foreign pathogens, pose a significant challenge to successful Tx. Several types or rejections exist, ranging from hyperacute and acute to chronic, all representing not only different rates of response but, also, different pathophysiological mechanisms in which both cellular and humoral immunity are involved. Despite constantly increasing the knowledge and broadened opportunities in immunosuppressive regimens, the full view of the immune response against allografts is still not fully understood, and the survival of transplanted organs remains time-limited [7].

**Figure 2:**
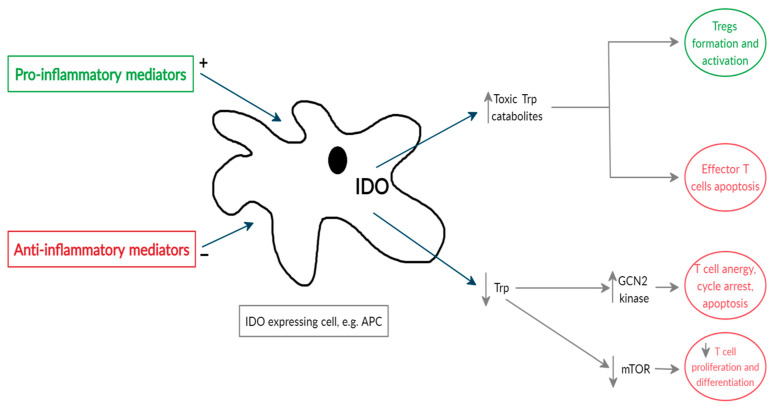
Simplified scheme of IDO activity in immune regulation. IDO is expressed in various cells, such as APCs, fibroblasts, endothelial, epithelial, smooth muscle cells, etc., in response to proinflammatory mediators. Due to IDO activity, decreased levels of tryptophan activate the GCN2 kinase, which leads to T-cell anergy, T-cell cycle arrest and apoptosis. The deactivated mTOR pathway results in suppressed T-cell proliferation and differentiation. Increased levels of toxic tryptophan-kynurenine pathway metabolites induce effector T-cell apoptosis and promote Treg formation and activation. Abbreviations: Trp: tryptophan, IDO: indoleamine-2,3-dioxygenase, APCs: antigen-presenting cells, GCN2: general control nonderepressible 2, mTOR: mammalian target of rapamycin and Tregs–regulatory T cells.

IDO is probably the most thoroughly investigated tryptophan-kynurenine pathway enzyme, which acts as a natural tolerogenic factor regulating immune responses (Figure 2).

IDO is expressed in several immune cells, such as monocytes, macrophages, dendritic cells (DCs) and microglia, as well as fibroblasts, endothelial cells, epithelial cells and smooth muscle cells [54]. Interferon (IFN)-γ is known as one of the main stimuli that promotes IDO synthesis at the transcriptional level [65]. Other inflammatory mediators capable of enhancing IDO activity—however, mostly to a lesser degree—include IFN-α; IFN-β; tumor necrosis factors (TNF)s; interleukins (e.g., IL-1β, IL-2, IL-6 and IL-27); CD40 ligand; prostaglandins and lipopolysaccharides (LPS) [66,67,68]. The aforementioned mediators activate specific receptors, such as aryl-hydrocarbon receptor (AhR), Toll-like receptors (TLRs), interferon-β and γ receptors and tumor necrosis factor receptors (TNFR), which subsequently promote intracellular signaling pathways, leading to IDO upregulation. Similarly, anti-inflammatory cytokines, such as IL-4, IL-10 and tumor growth factor (TGF)-β, were found to diminish the expression of IDO, confirming that the IDO status directly depends on the balance of pro- or anti-inflammatory responses [20,68]. The production of IDO was also found to be promoted by the engagement of CD80 (B7.1) and CD86 (B7.1) located on the surface of DCs with cytotoxic T-lymphocyte-associated antigen 4 (CTLA4) or CTLA4-Ig fusion protein [69,70]. The fusion of CD28 situated on T cells with CD80/86 on DCs is necessary for naïve T-cell activation. Endogenous CTLA4 expressed in activated conventional T cells and T-regulatory cells (Tregs) or exogenous CTLA4Ig (used as immunotherapy drug) are known to counterbalance CD28 in T cells due to a higher affinity for B7 molecules, resulting in the deactivation of the immune response [70,71,72,73]. It has been demonstrated that IDO inhibitor 1-MT abrogates a positive CTLA4Ig effect on the prolonged survival of mice pancreatic islet allografts [70]. However, recent data from a multicenter sub-study of the phase II trial showed that Betalacept, a second-generation CTLA4-Ig fusion protein, was not able to induce detectable IDO activity in human liver Tx recipients. Similarly, Betalacept did not have any effect on IDO expression in human dendritic cells in vitro [74].

As mentioned before (compare Figure 1), IDO, as the first enzyme in the tryptophan-kynurenine pathway, determines the local levels of tryptophan and tryptophan-kynurenine pathway metabolites. Tryptophan depletion increases the amount of free transfer RNA in T cells, which activates the GCN2 stress kinase pathway, leading to T-cell anergy and cell cycle arrest at the mid-G1 phase, thus sensitizing T cells to apoptosis [17,20,46,75]. Additionally, IDO significantly reduces the macrophage activity through the induction of apoptosis via the GCN2 kinase pathway and inhibition of inducible nitric oxide synthase (iNOS) expression, and this effect is also more related to tryptophan starvation rather than increased concentrations of tryptophan metabolites [76]. Moreover, it has been found that decreased levels of tryptophan may inhibit the mammalian target of the rapamycin (mTOR) pathway, resulting in the subsequential blockade of the translation process in T cells [77]. However, not all studies have confirmed that mTOR reacts to tryptophan depletion [46,75]. The theory of “tryptophan starvation” was also supported by in vitro experiments that showed that T-effector cells and macrophages may be reactivated in IDO-overexpressing cultured cells by adding an excessive amount of tryptophan [75,76].

On the other hand, active tryptophan metabolites kynurenine, 3-HK and 3-HAA, generated following IDO and other tryptophan-kynurenine pathway enzyme activity, promote oxidative stress and have a direct cytotoxic effect on T-effector cells, as well as suppress their proliferation by inducing apoptosis. Moreover, by inducing the apoptosis of T-helper 1 (Th1) cells, kynurenine metabolites are able to shift the balance between Th1 and Th2 towards Th2 and stimulate the formation of Tregs [20,78]. Kynurenine metabolites are also agonists of AhR, which plays an important role in Th17 cell differentiation and promotes the generation of Tregs [79,80,81]. Among the tryptophan metabolites produced downstream in the kynurenine pathway, 3-HAA appears to be the most potent immune regulator [82,83,84]. It has been previously revealed that 3-HAA induces the T-cell apoptosis activating PDK1 kinase [85] and caspase 8 [86]. Its dimerized form, cinnabarinic acid, produced in oxidant conditions was found to activate AhR as well [87]. A recent study also showed that 3-HAA engages nuclear coactivator 7 (NCOA7), expressed in dendritic cells, which increases the kynurenine-driven transcriptional activity of AhR. This combined mechanism of 3-HAA and kynurenine is important for the DC-mediated induction of Treg cells [88].

Even though the impact of IDO and the tryptophan-kynurenine pathway on cellular immune mechanisms has been widely investigated, much less is known about the humoral alloimmune response, which plays a role in Tx as well [89]. It has been reported that the B-cell-intrinsic promotion of IDO 1 via activation by Toll-like receptor ligands or B-cell receptor crosslinking is a key mechanism, reducing the production of antibodies against T-cell-independent antigens [90]. Another study, however, showed that IDO 2 expression in B cells increases the humoral autoimmunity by supporting the cross-interaction between reactive T and B cells [91]. An in vitro model by Sounidaki et al. recently demonstrated that the IDO inhibitor 1-metyltryptophan (1-MT) enhanced the humoral alloimmune response, demonstrating that IDO is also a possible inhibitor of humoral rejection mechanisms. However, the lack of effects from the AhR inhibitor and GCN2 kinase activator in these experiments suggests that molecular pathways that are responsible for the IDO effect on humoral alloimmunity differ from those participating in cellular alloimmunity inhibition, demanding further investigation [92].

Despite similar enzymatic function, in the field of immune regulation, TDO has been studied in a much lesser extent than IDO. Nevertheless, at the beginning of the current century, the TDO role in murine fetal tolerance was proposed [93,94]. An experimental study by Schmidt el al. revealed that TDO, similarly to IDO, is also capable of inhibiting the growth of bacteria, as well as restricting alloantigen-induced T-cell activation and inhibiting IFN-γ production. These effects were inhibited by additional tryptophan supplementation [95]. Another aspect, suggesting that TDO participates in the regulation of immune responses after Tx, is its promotion by glucocorticoids, which are included in classical immunosuppressive regimens [32]. Additionally, TDO has been detected in different tumors, such as melanomas, hepatocellular or bladder carcinomas, and its significance in tumor immune escape processes has been investigated [17,96].

KMO, as an enzyme standing at a cross-point of the “pro- and anti-inflammatory” or “oxidant and antioxidant” branches of the tryptophan-kynurenine pathway, has also gained interest in the research of immune regulation. The inhibition of KMO results in increased levels of the tolerogenic and anti-inflammatory metabolite KYNA, which diminish inflammation by activating AhR, reducing TNF expression in monocytes, IL-4 secretion by natural killer (NK) cells and IL-23 formation in DCs [17,20].

Due to the aforementioned immunomodulatory properties and the natural capability to downregulate allogeneic immune responses, the tryptophan catabolism via the kynurenine pathway has gained interest as an attractive therapeutic target in Tx. The in vivo studies investigating the role of enzymes and metabolites of the tryptophan-kynurenine pathway in immune regulation in solid organ Tx are summarized in Table 2 and discussed in detail below.

### 4.1. Heart

The impact of IDO gene delivery via an adenoviral vector directly into cardiac allografts has been investigated in several animal experimental models [97,98]. Li et al. reported that the intracoronary administration, as well as intramyocardial injection, of an adenoviral vector encoding for IDO cDNA (Ad-IDO) resulted in significantly prolonged cardiac graft survival in rats. Similarly, IDO gene transfer, together with a short course of low-dose Cyclosporine A (CsA), was more efficient than CsA alone. Interestingly, almost all the cells expressing IDO were cardiomyocytes. Histological examinations also revealed less cardiac infiltration by monocytes, macrophages and T cells (CD4+ and CD8α+), which indicates an attenuated rejection process [97]. These findings were confirmed by Yu et al., showing that intramyocardial Ad-IDO injection prolonged the cardiac allograft survival in mice [98]. Both studies reported significantly reduced mRNA transcript levels for proinflammatory cytokines and chemokines in Ad-IDO grafts [97,98]. However, despite the delayed rejection, Yu et al. showed a similar cellular infiltration in Ad-IDO-treated grafts as in control grafts, which emphasizes a probable capability of IDO to modify the function rather than number of alloreactive T cells. Ad-IDO grafts also showed a significantly higher proportion of Tregs [98]. As IDO-transduced DCs demonstrated the ability to inhibit allo-specific T-cell proliferation in vitro [97], IDO-overexpressing DC injection into the recipient before cardiac Tx has also been investigated [99,100,101]. Decreased INFγ and increased IL-10 intra-graft transcription-induced CD4+-cell apoptosis, greatly diminished histopathology changes and significantly prolonged graft survival have been reported after pre-Tx IDO-overexpressing DC transfusion. Interestingly, IDO was also mainly expressed in cardiomyocytes [99]. Ly et al. used a combined recipient preconditioning with a pre-Tx infusion of Ad-IDO-transfected DCs and a post-Tx infusion of CD40L mAb, which also acts via IDO upregulation [100]. They reported significantly lower serum INF-γ and IL-2 expression, an increased apoptosis of peripheral CD3+ T cells and decreased creatine kinase (CK) and lactate dehydrogenase (LDH) levels, referring to the improvement of allograft function after cardiac Tx. More significant effects were achieved by using the combination therapy of IDO-overexpressing DCs and CD40L mAb compared to IDO-overexpressing DCs alone [100]. The combination treatment with IDO-transfected DCs and tryptophan metabolites was significantly more effective in allograft rejection delay and survival prolongation in comparison to each of these strategies separately [101]. Among tryptophan metabolites, contrary to AA and QUIN, 3-HAA, 3-HK and L-kynurenine showed the best ability to inhibit the proliferation of unstimulated spleen-derived T cells by the induction of apoptosis in vitro. A single 3-HAA and allogeneic bone marrow-derived DC injection of recipient rats resulted in significantly prolonged cardiac graft survival and lowered the pathological grade of rejection, while a 3-HAA injection alone had only minimal protective effects. The most important finding of this experiment was that 3-HAA-suppressed T cells could not be restimulated by donor-specific DCs [83]. Another two studies by He et al. showed that IDO-overexpressing bone marrow mesenchymal stem cells [102] or exosomes derived from IDO-overexpressing bone marrow mesenchymal stem cells [103] injected intravenously 48 h after allogeneic heart Tx of rats similarly resulted in significantly improved graft function. This was demonstrated by determination of the ejection fraction and left ventricular fractional shortening, as well as decreased graft damage and infiltration by inflammatory cells compared to mycophenolate mofetil-treated or untreated animals. The authors reported significantly lower serum levels of IL-1α, IL-4, IL-1β, IL-2, IFN-γ and IL-18 and increased levels of IL-10, TGFβ1, TGFβ2 and TGFβ3 in both models. The flow cytometry of isolated cells of rat spleens showed significantly lower expressions of CD40, CD86, CD80, MHC-II, CD45RA and CD45RA+CD45RB and a higher expression of CD274 and a higher proportion of Tregs than in the control groups [102,103]. A proteomic analysis demonstrated that exosomes secreted by IDO-overexpressing mesenchymal cells contained significantly upregulated immunoregulatory protein FHL-1 [103]. Importantly, the tolerogenic state, achieved by direct IDO gene transfer to cardiac cells or by IDO-overexpressing DC infusion, is allograft-specific and not only limited to the local milieu but is rather systemic, as shown by the increased survival of secondary implanted skin allografts that were genetically identical to implanted hearts [98,101].

### 4.2. Lungs

The survival rates after lung Tx remain significantly lower compared to other solid organ Tx, mainly due to the more active immune response to lung allografts [104]. Lung interstitial and peribronchial tissue are well-supplied with antigen-presenting cells (APCs), such as DCs, monocytes and macrophages, that can strongly stimulate or suppress the immune response. There is evidence that IDO is significantly upregulated in lung tissue during microbial infections after allogeneic hematopoietic stem cell Tx and under other inflammatory conditions [105,106,107]. Importantly, it has been found that IDO is induced and the tryptophan-kynurenine pathway is promoted not only in antigen-presenting but, also, in nonimmune pulmonary cells, such as epithelial cells, which is important in the local protection of lung tissue from collateral damage [105,106,107]. Therefore, among a bunch of therapeutic strategies, targeting the tryptophan-kynurenine pathway has also been investigated in several in vivo animal models of lung Tx. Swanson et al. were the first to report that systemic Ad-IDO vector instillation into the donor’s organism before lung Tx results in an abrogated delayed-type immune response and lowers the histopathological grade of allograft rejection [108]. In another study, the intratracheal nonviral delivery of IDO preserved the allograft function, which was reflected by a significantly reduced peak airway pressure and increased PaO_2_ levels in IDO-transfected grafts. In vitro experiments revealed that the protective effect of IDO is achieved not only through T-cell inhibition but, also, through the enhancement of the local antioxidant defense system when the graft faces the burden of oxidants [109]. The possible therapeutic role of IDO was also assessed in a model of chronic lung allograft injury using IDO gene delivery via the “Sleeping beauty“ transposon, which, differently to most viral vectors, is capable of promoting a long-lasting expression of the desired gene in target tissues. IDO-positive lung cells inhibited the TGFβ-mediated proliferation of fibroblasts and excessive accumulation of collagenous tissue, which resulted in reduced graft fibrosis, preserved bronchus-alveolar architecture and significantly improved the function of the preconditioned lung allograft [110]. Further investigating the mechanism of the tryptophan metabolism-induced tolerance of lung allografts, the same group reported that, despite IDO overexpression, the number of allograft-infiltrating CD8 T cells is still higher than in normal lungs or isografts, revealing that IDO effects in immune modulation may be incomplete. Nevertheless, a significant reduction of T-cell cytotoxicity by disturbance of the granule perforin and granzyme exocytosis was demonstrated. T cells from treated allografts produced significantly less IL-2 and TNF-α, while the INF-γ production remained obvious. Interestingly, the impaired function of complex I of the electron transport chain in CD8+ cell mitochondria was revealed, while complexes II-IV were still intact. This may be the reason for the survival of CD8+ cells in a high-IDO environment [111]. In another study, 3-HAA delivery seven days after lung Tx showed similar immune-protective effects as donor preconditioning by IDO gene transfer. Moreover, comparable results in GCN2 gene knockout mice were demonstrated, strengthening the theory that the upregulation of tryptophan metabolites may be more critical in immune regulation than tryptophan depletion. The study found that high IDO/3-HAA levels inhibit T cells due to the impairment of calcium signaling via the T-cell receptor (TCR)/Ca2+ signaling pathway [84]. Finally, in recent experiments, the IDO gene was transferred into tissue-engineered lung allografts, which were constructed from decellularized rat lungs, differentiating medium and rat bone marrow mesenchymal stem cells. IDO gene transfer into such composites allowed to achieve tolerogenic status by the reduction of inflammatory cytokines levels and upregulation of regulatory T cells. Thus, IDO overexpression may be considered as one of the possible methods allowing to go one step further in setting up a nonimmunogenic lung tissue construct and to overcome the problem of graft shortage [112].

### 4.3. Liver

It is well-known that, unlike other solid organs, fully allogeneic liver grafts are accepted spontaneously in most mice strains combinations. Miki et al. were the first investigators who demonstrated that, unless IDO mRNA is not expressed in the mouse liver in physiological conditions, it is induced after allogeneic liver Tx, and the inhibition of IDO activity and tryptophan metabolism leads to the rejection of otherwise spontaneously accepted liver grafts [113]. The IDO gene was found to be significantly upregulated in allogeneic spontaneously accepted liver tissue, while naïve and syngeneic livers express IDO mRNA. Moreover, IDO was expressed only in liver antigen-presenting cells such as dendritic or Kupffer cells, while hepatocytes lacked an IDO signal [114,115]. Several studies suggested that TDO, which is a liver-specific tryptophan-kynurenine pathway enzyme, may also play a role in immune response regulation after allogeneic liver Tx [95,116]. However, in studies by Lin et al., TDO mRNA was found to be downregulated in allogeneic livers, implicating the probability that TDO does not participate in immune modulation, but rather, the depletion of this enzyme induces hepatocyte damage [114,115]. The reinfusion of IFN-γ-treated dendritic cells, showing vastly enhanced IDO mRNA expression, into liver-transplanted rats resulted in attenuated allogeneic liver graft rejection. The tryptophan levels decreased during the first week after Tx, followed by a continuous increase from day seven [117]. Contrary to these findings, Laurence et al., by inhibiting IDO activity by means of the IDO inhibitor 1-metyltryptophan (1-MT), in allogeneic liver-transplanted rats for the first week after Tx, did not cause acute rejection [118]. The same group confirmed their findings in another experiment with preconditioned liver donor rats with an IDO-containing recombinant adeno-associated virus vector; this intervention did not result in the prevention of liver allograft rejection signs, despite the confirmed IDO activity in vivo [119]. Another interesting animal allogeneic liver Tx experiment was conducted using N-(3′,4′-dimethoxycinnamonyl) anthranilic acid (3,4-DAA), which was injected intraperitoneally into recipient rats immediately after Tx. A synthetic derivative of the natural tryptophan-kynurenine pathway branch metabolite anthranilic acid, 3,4-DAA, has been approved in Japan as an antiallergic drug and is known for its ability to increase IDO expression. This study demonstrated that 3,4-DAA treatment resulted in attenuated liver allograft injury through the decreased TNF-α and IFN-γ, as well as increased IL-10 inflammatory signaling, while the IDO inhibitor 1-MT abrogated this effect. Significantly increased L-kynurenine levels were observed in 3,4-DAA-treated rats [120].

### 4.4. Kidneys

Cook et al. were the first to demonstrate the immunomodulatory effects of reduced local tryptophan concentrations and increased tryptophan metabolites in renal allografts. They suggested that the early reduction of cellular rejection in allografts is associated with the TGF-β-mediated mechanism, while long-term tolerance is achieved via the IDO-induced regulation of reactive T cells [121]. Oppositely, the pre-Tx injection of immature IDO knockout dendritic cells to renal allograft recipient mice overturned the tolerogenic effects of normal immature dendritic cells. This resulted in a partial reverse of the reduction of T-cell proliferation, decreased proportions of CD4+CD25+Foxp3+ cells and increased cellular infiltrations in grafts, as well as upregulated IgG production, IL-2 and IFN-γ expression and, most importantly, deteriorated allograft survival and function, as confirmed by elevated serum creatinine levels [122]. Renal tubular epithelial cells, representing 75% of the renal parenchymal cells, are the main targets of the T-cell-mediated immune response. There is evidence that these cells have immunomodulatory capacities similar to mesenchymal stem cells or antigen-presenting cells and express IDO when stimulated by IFN-γ and TNF-α [51]. Tubular epithelial cells were found to have the ability to inhibit CD4+ and CD8+ T-cell proliferation. However, the inhibition of IDO by 1-MT only partially restored the activity of effector T cells. Importantly, it has been revealed that the tubular epithelial cell immunosuppressive ability is cell–cell contact-dependent; therefore, they are not able to influence T cells, which infiltrate the renal interstitial compartment and are not directly exposed to epithelial cells [123]. Mesenchymal stem cells are known for their natural ability to restrain the proliferation and activation of T, B and natural killer cells, as well as to inhibit inflammation. IDO, produced by mesenchymal stem cells, has been proven to be crucial for the promotion of Treg generation and improvement of graft survival in rat kidney Tx models [124]. Unless the injection of high doses of mesenchymal stem cells revealed positive effects in preventing acute allograft rejection and tolerance induction [125], high doses of these cells may increase the risk for other complications like infections, thrombotic microangiopathy or organ infarction [126]. He et al. injected IDO-expressing mesenchymal stem cells into renal allograft recipients, resulting in significantly prolonged graft survival and enhanced donor-specific tolerance by inducing the production and improving the function of antigen-specific Tregs in a rabbit model. Importantly, low doses of IDO-mesenchymal stem cells were sufficient to achieve tolerogenic effects [127].

The group of Vavrincova-Yaghi investigated the role of IDO gene therapy in kidney allograft preconditioning after organ retrieval. IDO transgene delivery directly into the kidney graft resulted in a significantly attenuated increase in plasma creatinine and improved allograft function, as well as reduced levels of kidney injury markers KIM-1 and alpha smooth muscle actin (α-SMA). Significantly reduced inflammation and upregulated Treg cell markers were found in IDO-preconditioned kidneys, which was accompanied by preserved tubular morphology and reduced interstitial pre-fibrosis. Despite the locally increased transgene expression in the renal interstitium, no significant difference in the systemic kynurenine/tryptophan ratio was found in IDO-transfected rats, suggesting that the effect of IDO overexpression remained local [128]. Interestingly, human IDO mRNA was absent in IDO-preconditioned transplanted kidneys at 12 weeks after Tx, which shows again that adenoviral transgene expression is limited in time. However, several clinical improvements, like the gaining of body weight, increase of systolic blood pressure or prevention of proteinuria, were still observed within the 12-week period in IDO-treated animals [129]. It has been previously found that tryptophan metabolism to kynurenines induced by IDO expressed in endothelium leads to the relaxation of arterial vessels via the adenylate and guanylate cyclase pathways during inflammatory conditions and sepsis [130]. Vavrincova-Yaghi et al. found reduced angiotensin-converting enzyme mRNA expression in IDO-preconditioned renal grafts, which could be another favorable mechanism through which IDO may reduce arterial blood pressure [129]. Moreover, kidney graft preconditioning with IDO in a long time period protected against transplant vasculopathy, which is one of the most important pathophysiologic elements in chronic transplant rejection, as proliferated neointima and narrowed vascular lumen lead to hypoperfusion, subsequent graft fibrosis and chronic transplant dysfunction [129]. A murine model of heterotopic aortic Tx revealed that the daily administration of various doses of antioxidant sodium sulfite blocked tryptophan breakdown through the kynurenine pathway and resulted in significant vasculopathy, as well as increased levels of α-SMA, vascular cell adhesion molecule (VCAM)-1 and P-selectin. These findings proved the role of tryptophan metabolism in chronic transplant rejection and highlighted the challenges associated with antioxidant treatments, as they apparently may have ambivalent consequences [37].

In a recent porcine kidney allo-Tx study, the allogenic immune response significantly promoted the IDO gene and proinflammatory cytokines expression and, additionally, suppressed another tryptophan kynurenine pathway enzyme KMO [131]. As 3-HK, a KMO-catalyzed reaction product, is known to be a more potent immunosuppressive catabolite than L-kynurenine [83], its loss due to KMO suppression may explain the inability of elevated allograft IDO activity to fully inhibit rejection [131] and give some implications about KMO or KAT as another potential therapeutic target of immune modulation.

### 4.5. Pancreas

Unfortunately, there are no studies, examining the immunoregulatory properties of tryptophan-kynurenine pathway enzymes and metabolites in the setting of whole pancreas Tx so far. Nevertheless, these questions have been investigated in several experimental animal models of pancreatic islet Tx. Alexander et al. found IDO gene transfer via the adenoviral vector to pancreatic cells resulting in significantly prolonged graft survival, increased amounts of insulin-positive β cell mass and the inhibition of T-cell proliferation [132]. Avoiding the potential harmful effects of the adenovirus on pancreatic islets [133,134], Jalili et al. created a three-dimensional islet graft by embedding allogeneic mouse islets and IDO-transduced fibroblasts within a collagen gel matrix. Interestingly, IDO was still expressed in the graft, whereas T-cell infiltration was not detected 40 days after Tx. The inclusion of IDO-overexpressing fibroblasts into this composite model also promoted Treg upregulation in graft-draining lymph nodes, generated anti-inflammatory cytokine profiles, inhibited the production of donor specific alloantibodies and significantly increased the number of insulin-producing cells. However, this effect was only observed until the fifth week after graft implantation, proving that IDO expression and its mediated immunosuppression was transient [135]. By using a lentiviral IDO vector combined with a protease-resistant composite scaffold, the immunosuppression could be prolonged for two weeks. However, a gradual decline of IDO expression and the respective immunosuppression remained inevitable. The authors emphasized that immune stability is maintained until a sufficient amount of Tregs is present [136]. The same group proposed a pancreatic islets xenogeneic Tx model. They implanted a collagen matrix-containing rat islet and adenoviral-transduced IDO-expressing mouse fibroblasts into recipient mice. The results indicated that IDO suppresses macrophage and T-cell infiltration into the graft, as well as inhibits iNOS expression by macrophages, which is extremely important in xenogeneic Tx, as macrophage activity is mainly responsible for xenograft rejection. Interestingly, in vitro experiments showed that a tryptophan deficiency, rather than increased levels of tryptophan metabolites, was responsible for macrophage viability reduction and apoptosis induction [76]. Another study investigated, in a systemic approach, the effects of intraperitoneal-injected IDO-expressing fibroblasts in recipients. This treatment delayed the rejection of pancreatic islet allografts culminating in a part of recipients, even reaching immune tolerance and leading to the assumption that the systemic delivery of IDO-overexpressing fibroblasts may be more efficient than local applications, probably due to the possibility to inject more cells [137].

### 4.6. Small Bowel

One of the most challenging features of intestinal allografts is the extremely high immunogenicity compared to other organ grafts; proper immune regulation is still a major obstacle. A murine small bowel Tx model revealed that a recipient treatment with IDO-transfected DCs, intravenous 3-HAA or a combination of these approaches results in a significantly prolonged graft survival, reduced inflammation and diminished morphological graft distortion. Interestingly, IDO-DCs seem to have stronger immunosuppressive effects than 3-HAA. Despite that in vitro 3-HAA enhanced the immunosuppressive effect of IDO-DCs when used in combination, in vivo, no significant improvement in allograft survival was observed compared to the IDO-DC treatment alone. Despite the potential therapeutic effect of IDO in intestinal Tx, the increased risk of intestinal tumors due to IDO activity should be taken into account [82].

To sum it up, targeting tryptophan metabolism via the kynurenine pathway seems an attractive strategy for immune regulation in solid organ Tx via mechanisms decreasing the inflammatory changes, such as IRI and both acute and chronic rejection. While human recipient preconditioning or post-Tx treatment with IDO gene therapy might still raise concerns, especially due to unknown long-term systemic effects and their related dangers, intensifying the investigation on novel organ preservation techniques, such as normothermic or sub-normothermic machine perfusion, will potentially provide an attractive platform for gene therapy by direct IDO gene transfer to graft cells or IDO-transfected APC delivery directly to the organ. Nevertheless, the current evidence is limited to in vitro experiments and animal models, revealing that there are still several challenges to overcome. Most of the studies only succeeded in delaying the acute rejection, but absolute allograft tolerance was usually not achieved, and allograft survival remained time-limited. This means that the use of more stable vectors in gene therapy, multiple dosage recipient treatment after Tx or a combination with conventional immunosuppressive therapy may be necessary. There is still also a lack of evidence regarding the role of tryptophan and its metabolites in the regulation of humoral alloimmunity, which is also an important player in the organism’s general immune response against allografts [89,92]. The current experimental evidence still lacks information about the IDO gene therapy effects on long-term graft conditions and functions, as well as systemic consequences. For example, the creation of a tolerogenic environment by enhancing the tryptophan breakdown carries a risk of the development and expansion of malignant cells, which could potentially be one of the biggest drawbacks of IDO gene therapy [17]. Therefore, further investigation is still necessary to answer the remaining questions regarding the effectiveness and safety of targeting the tryptophan-kynurenine pathway, especially using gene therapy, for post-Tx immune regulation.

**Table 2:** Immunomodulatory effects of targeting the tryptophan-kynurenine pathway in animal models of solid organ transplantation (Tx).

Reference	Experimental Model	Treatment/Intervention	Outcomes
**Heart**			
Li et al. [97], 2007	Rats model of heart Tx	IDO gene transfer into the donor heart via adenoviral vector by an intracoronary infusion or intracardiac injections of vector-containing (10^10^ PFU) solution immediately before Tx.	↓mRNA levels of IFN-γ, TNF-α, TGF-β, IL-1β; ↓graft infiltration with monocytes, macrophages, T effector cells; ↑graft survival.
Yu et al. [98], 2008	Mice model of heart Tx	IDO gene transfer into the donor heart via adenoviral vector by intracardiac injections of vector-containing (10^10^ PFU) solution immediately after Tx.	↓mRNA levels for IL-2, IL-17, IFN-γ; ↑proportion of Tregs, no difference in leucocyte infiltration; delayed rejection, ↑graft survival.
Dai et al. [83], 2009	Rats model of heart Tx	Single injection of allogeneic bone marrow dendritic cells+3-HAA for recipient animals 7 days before receiving the graft.	↓mRNA levels for IL-2, IL-17, IFN-γ; ↑proportion of Tregs, no difference in leucocyte infiltration; delayed rejection, ↑graft survival.
Li et al. [99], 2016	Mice model of heart Tx	10^6^ donor’s DCs transfected with IDO gene via adenoviral vector, infused intravenously 3- and 1- day before Tx.	↓mRNA levels of IFN-γ, ↑ IL-10; ↑ CD4+ T cells apoptosis; ↓histopathological changes; ↑ graft survival.
Lv et al. [100], 2018	Mice model of heart Tx	10^6^ donor’s DCs transfected with IDO gene via adenoviral vector, infused intravenously 5- and 3- days before Tx, combined with 250-μg/d CD40L mAb infused intravenously at 0, 1, 2 and 4 days after Tx.	↓serum levels of INF-γ and IL-2; ↑apoptosis of peripheral CD3+ T cells; ↓serum CK and LDH levels. More significant effect of combined therapy than IDO+ DCs alone.
He et al. [103], 2018	Rats model of heart Tx	IDO 1-overexpressing (lentivirus-transfected) bone marrow mesenchymal stem cells exosomes injected intravenously (1 mL; 20 mg/mL) 48 h after heart Tx.	↑graft function (↑ejection fraction and left ventricular fractional shortening on days 2, 4 and 7); ↓graft infiltration by inflammatory cells; ↓serum levels of IL-1α, IL-4, IL-1β, IL-2, IFN-γ, IL-18; ↑levels of IL-10, TGFβ1, TGFβ2 and TGFβ3; ↓expression of CD40, CD86, CD80, MHC-II, CD45RA and CD45RA+CD45RB and ↑expression of CD274; ↑proportion of Tregs in spleen; ↑immunoregulatory protein FHL-1. miR-540-3p was the most highly upregulated microRNA, and miR-338-5p was the most highly downregulated microRNA in IDO+ exosomes.
He et al. [102], 2020	Rats model of heart Tx	10^6^ donor bone marrow mesenchymal stem cells transfected with IDO via the lentivirus injected intravenously 48 h after heart Tx.	↑graft function (↑ejection fraction and left ventricular fractional shortening on days 2, 4 and 7 post-Tx; ↓graft infiltration by inflammatory cells; ↓hemorrhage, edema, and myocardial damage; ↓serum levels of IL-1, IL-4, IL-2, IFN-γ, IL-18 and ↑ IL-10, TGFβ1, TGFβ2 and TGFβ3. ↓expression of CD40, CD86, CD80, MHCII, and CD45RA+CD45RB, and ↑ CD274; ↑Tregs.
Li et al. [101], 2020	Mice model of heart Tx	Donors DCs transfected with IDO gene via adenoviral vector combined + tryptophan catabolic products infused intravenously to recipient 3 days before Tx.	↓IL-2, IFN-γ and TFN-α, ↑IL-10 mRNA and protein expression; ↑CD4+ T cells apoptosis; ↓histopathological injury level; ↑allograft survival. Effect more significant with combined therapy than either of therapies alone.
**Lungs**			
Swanson et al. [108], 2004	Rats model of lung Tx	IDO gene transfer into the donor lungs via adenoviral vector by instillation of vector-containing (10^7^ PFU) solution 24 h before Tx.	↓delayed-type hypersensitivity responses to donor antigens, ↓graft rejection histopathological grade.
Liu et al. [109], 2006	Rats model of lung Tx	hIDO gene transfer via PEI carrier. 0.2 mL of transfection solution containing 20 mcg of plasmid DNA delivered to donor rat lung via an intratracheal catheter 24 h before Tx.	↓peak airway pressure, ↑PaO_2_; ↓level of acute cellular rejection and preserved graft architecture in histopathology; ↓CD3 and MPO-positive cells infiltration; ↓necrosis and apoptosis of lung cells, ↓ intracellular ROS formation.
Liu et al. [110], 2006	Rats model of lung Tx	Sleeping beauty transposon mediated hIDO gene delivery (50 mcg of plasmid DNA) to donor rat lung via an intratracheal catheter 24 h before Tx.	↓peak airway pressure, ↑PaO_2_; inhibition of TGFβ mediated proliferation of fibroblasts; ↓graft fibrosis; preserved bronchus-alveolar architecture.
Liu et al. [111], 2009	Rats model of lung Tx	Sleeping beauty transposon mediated hIDO gene delivery. 450 mcg of plasmid DNA delivered to donors intravenously 24 h before Tx.	↓acute cellular rejection grade and graft injury; ↓peak airway pressure, ↑PaO_2_. Impaired function of complex I of the electron transport chain in mitochondria, inhibited cytotoxic function of lung infiltrating T cells; ↓production of IL-2 and TNF-α, but remaining production of IFN-γ (in CD8+ T cells isolated from lung allografts).
Iken et al. [84], 2012	Mice model of lung Tx	hIDO gene transferred to donor lung via non-viral PEI carrier; Intraperitoneal injections of 250–350 mg/kg 3-HAA for 7 days.	↓acute cellular reduction grade and graft injury; ↓peak airway pressure, ↑PaO_2_; ↑graft survival due to T cell inhibition: (a) Impaired TCR activation through the interruption of intracellular calcium (Ca21) and of the TCR/Ca21 signaling pathway; (b) ↓levels of proinflammatory cytokines and chemokines (evident ↓IL-2, IL-4 IL-6, IL-5, IL-13, 50% ↓IFN-γ, TNF-α, IL-12, no change of IL-10, IL-17) in allografts; Maintenance of naïve T cells rather than the generation of effector memory T cells.
Ebrahimi et al. [112], 2016	Rats model of engineered lung tissue Tx	IDO transduction to the engineered Lung Tissue via IDO expressing lentivirus.	↓histopathological score of acute rejection, ↓TNF-α and IFN-γ gene expression, ↑level of FOXP3 expression (↑Tregs), ↓RANTES.
**Liver**			
Laurence et al. [119], 2009	Rats model of liver Tx	IDO gene transfer via recombinant adeno-associated virus vector. Donors pretreated with 10^13^ Vg of the recombinant virus (rAAV2/8-LSP1-rIDO) by infusion into the portal vein 2 weeks prior to Tx.	Despite confirmed in vivo IDO activity, rAAV2/8-LSP1-rIDO failed to prevent liver allograft rejection (recipient and graft survival, histological features did not differ between groups).
Sun et al. [120], 2011	Rats model of liver Tx	200 mg·kg^−1^·day^−1^ of 3,4-DAA injected intraperitoneally immediately after surgery.	↓serum ALT; ↓level of injury in histopathology; ↓TNF-α, IFN-γ, ↑IL-10 mRNA expression.
**Kidney**			
Vavrincova-Yaghi et al. [128], 2011	Rats model of kidney Tx	IDO gene transfer to donor kidneys via RGD-modified adenovirus. Solution with 4 × 10^11^ viral particles infused into renal artery of retrieved kidney and incubated for 20 min in 4 °C saline.	↓increase in plasma creatinine; ↓the interstitial infiltration of CD8+ T cells and macrophages; ↓expression of SMA- α and KIM-1 mRNA; ↓expression of IL-2, IL-17, TGF-β mRNA, ↑levels of foxp3 mRNA (↑Tregs).
He et al. [127], 2015	Rabbits model of kidney Tx	Mesenchymal stem cells transfected with IDO gene via recombinant lentivirus. IDO-MSCs (2 × 10^6^ cells/kg) injected intravenously to recipient mice after Tx.	Induced donor specific allograft tolerance via ↓of CD4+CD25− T-cells, ↑of CD4+CD25+ Foxp3+ (Tregs), ↑ antigen-specific immune-suppressive functions of CD4+CD25+ Tregs, ↑CTLA-4 expression by CD4+CD25+ Tregs, stimulation of Treg cells to secrete IL-10 and TGF-β1; ↑graft survival, ↓acute rejection signs (↓serum creatinine levels, preserved normal histological graft structure).
Vavrincova-Yaghi et al. [129], 2016	Rats model of kidney Tx	IDO gene transfer to donor kidneys via RGD-modified adenovirus. Solution with 4 × 10^11^ viral particles infused into renal artery of retrieved kidney and incubated for 20 min in 4 °C saline.	In a long-time period (12 weeks) treatment protected against development of transplant vasculopathy, rise of systolic blood pressure and proteinuria; plasma creatinine did not reduce significantly; ↑expression of TGF-β and foxp3 mRNA; ↓expression of ACE mRNA.
**Pancreas**			
Alexander et al. [132], 2002	Mice model of pancreatic islets Tx	IDO gene transfected into pancreatic islets via recombinant adenovirus.	↑graft survival, ↓proliferation of T cells, ↑remaining insulin producing cells.
Jalili et al. [135], 2010	Mice model of pancreatic islets Tx	Composite three-dimensional islet grafts engineered by embedding allogeneic mouse islets and adenoviral-transduced IDO–expressing syngeneic fibroblasts within collagen gel matrix.	↓CD4+ and CD8+ effector T-cells infiltration at the graft site, ↑number of Treg cells in graft-draining lymph nodes; ↓gene expression of proinflammatory cytokines (IL-2, IL-17, CXCL9 and CXCL10), ↑gene expression of anti-inflammatory cytokines (IL-4, IL-10); delayed allo-specific antibody production, ↑viability of insulin secreting cells. Immunosuppressive effect limited to 5 weeks post-Tx.
Poormasjedi-Meibod et al. [76], 2013	Mice model of pancreatic islets xenoTx	Composite three-dimensional islet grafts engineered by embedding allogeneic rat islets and adenoviral-transduced IDO-expressing syngeneic mouse fibroblasts within the collagen gel matrix.	Well-preserved islet morphology, ↑number of insulin and glucagon expressing β cells, ↓number of infiltrating macrophages and CD3+ T cells, ↓iNOS expression.
Hosseini-Tabatabaei et al. [136], 2015	Mice model of pancreatic islets Tx	Composite three-dimensional islet grafts engineered by embedding allogeneic rat islets and lentivirus-transduced IDO-expressing syngeneic mouse fibroblasts within protease-resistant scaffold.	↑islet allograft survival (until 7 weeks); ↑population of foxp3+ Tregs at the graft site and graft-draining lymph nodes, ↓T-cell infiltration. Better-preserved functional β cell mass.
**Small bowel**			
Xie et al. [82], 2015	Mice model of small bowel Tx	IDO gene transfected to DCs via adenoviral vector. Three treatment groups: (A) 2 × 10^6^ IDO gene-transfected DCs injected intravenously immediately after Tx; (B) 120-mg/kg 3-HAA injected intravenously 7 days before Tx; (C) Both.	All treatment types ↓effector T cells and ↑Foxp3+ Tregs, ↓plasma pro-inflammatory cytokines (IFN-γ, IL-2) and ↑anti-inflammatory cytokines (IL-10, TGFβ), preserved histological graft structure. All strategies ↑graft survival compared to control, however IDO-DCs were more effective than 3-HAA. No significant difference in graft survival when used both.

Abbreviations: Tx: transplantation, (h)IDO: (human) indoleamine-2,3-dioxygenase, PFU: plaque-forming unit, IFN: interferon, TNF: tumor necrosis factor, TGF: tumor growth factor, IL: interleukin, 3-HAA: 3-hydroxyanthranilic acid, CD: cluster of differentiation, DCs: dendritic cells, CK: creatine kinase, LDH: lactate dehydrogenase, MHC: major histocompatibility complex, PEI: polymer polyethyleneimine, MPO: myeloperoxidase, TCR: T-cell receptor, foxp3: Forkhead box P3, RANTES: Regulated upon Activation, Normal T Cell Expressed and Presumably Secreted, Tregs: regulatory T cells, RNA: ribonucleic acid, DNA: deoxyribonucleic acid, ALT: alanine aminotransferase, 3,4-DAA: 3,4,-dimethoxycinnamoyl anthranilic acid, CTLA: cytotoxic T-lymphocyte-associated protein, ACE: angiotensin-converting enzyme, ROS: reactive oxygen species, PaO_2_: partial oxygen pressure, KIM: kidney injury molecule, SMA: smooth muscle actin and iNOS: inducible nitric oxide synthase, ↑: increased, ↓: reduced.

## 5. Diagnostic and Prognostic Role of Tryptophan and Its Metabolites in Solid Organ Tx

The assessment of graft quality, prediction of the early- and long-term graft outcomes and risk of post-Tx mortality of recipients is another important aspect in the field of solid organ Tx. The early detection of specific biomarkers of rejection, delayed graft function or primary nonfunction are also essential to identify patients at risk and put more effort on the follow-up and timely treatment in these cases. Tryptophan and its metabolites have been investigated as attractive diagnostic and/or prognostic tools in solid organ Tx (Table 3).

There is evidence that about half of recipients experience inflammatory conditions after kidney Tx, such as cellular or humoral graft rejections, bacterial, viral or mycotic infections, which increase the level of proinflammatory mediators and induce tryptophan catabolism by IDO [138]. Correspondingly, it has been suggested that low IDO activity and stable low serum levels of tryptophan catabolite kynurenine after kidney Tx are related to superior graft survival, as an immune balance has been reached [139]. Lahdou et al. [140] and Brandacher et al. [141], investigating adult kidney recipients, reported that the serum kynurenine/tryptophan ratio in the recipients without acute rejection drops to a level comparable with healthy non-transplanted subjects as early as in the first three weeks [141] or six months [140] after graft implantation. Interestingly, another group showed that stable pediatric kidney Tx recipients (no acute graft rejection and no infection) had significantly higher urine kynurenine/tryptophan ratios within the first month post-Tx compared to healthy children. This suggests that a slightly increased tryptophan catabolism may probably reflect the maintenance of the stable immunological/anti-inflammatory state after Tx [142]. Holmes et al. were the first to report that serum kynurenine levels significantly increase at five–seven days before biopsy-proved kidney allograft rejections, as well as in cases of viral or bacterial infections [143]. Brandacher et al. confirmed these results, showing that the serum and urine kynurenine/tryptophan ratios and kynurenine alone permit an accurate diagnosis of acute graft rejection. Importantly, changes of kynurenine/tryptophan were observed as early as one day after Tx, much earlier than the episode of rejection, and suggests a predictive role of tryptophan catabolism in transplanted patients. However, no significant changes in either levels of tryptophan, kynurenine or the kynurenine/tryptophan ratio were associated with infection episodes in the observed cohort [141]. Lahdou et al. demonstrated that recipients experiencing acute kidney graft rejection had significantly higher plasma kynurenine levels already prior to Tx compared to patients who had favorable early graft outcomes [140]. Although these early observations were limited to small cohorts [140,141,143], later larger retrospective and prospective studies confirmed rather than denied these results. In a large retrospective study, Kaden et al. demonstrated that kidney recipients with immediately functioning grafts show a significant decrease of serum kynurenine levels to almost normal values already at day five after Tx, and this normal course in kynurenine dynamics is changed by inflammatory events that activate IDO. The increased levels of kynurenine in the context of acute graft rejection in this study was dependent on the severity of rejection (the lowest increase in steroid-sensitive rejection and the highest one in vascular rejection). Moreover, the successful treatment of rejection resulted in decreased plasma kynurenine levels [144]. In a prospective study by Vavrincova-Yaghi et al., a rapidly increasing serum kynurenine/tryptophan ratio as early as the first day after Tx was associated with acute graft rejection [145]. Kim et al. detected five urinary biomarkers related to T-cell mediated kidney allograft rejection by using a metabolomics approach: a positive association was found with guanidoacetic acid, methylimidazoleacetic acid and dopamine and a negative one with 4-guanidinobutyric acid and L-tryptophan. Together, these metabolites predicted acute T-cell-mediated rejection with an accuracy of 87% for the training set; however, in the validation set, the accuracy decreased to 62.5% [146]. In another targeted metabolomics study that investigated urinary metabolites, kynurenine was found as one of the top 10 metabolites able to identify acute T-cell-mediated rejection in pediatric kidney Tx recipients [147]. On the contrary, Dharnidharka et al. did not observe such diagnostic and prognostic effects of urine tryptophan, kynurenine or the kynurenine/tryptophan ratio. However, they found that the increased serum kynurenine/tryptophan ratio, but not tryptophan or kynurenine alone, reflects episodes of acute rejection in children after renal Tx [148]. Interestingly, another metabolomics study, investigating metabolic changes in acute renal allograft rejection, reported converse results. Serum tryptophan was decreased and kynurenine increased in the group of patients experiencing no acute graft rejection in comparison to recipients who did. The authors explained this finding as a reflection of the increased IDO activity and its probable tolerogenic role for relieving acute rejection [149].

The role of kynurenine in the diagnosis of infectious events of kidney Tx recipients has also been demonstrated. In the case of cytomegalovirus (CMV) infection, elevated serum kynurenine levels were found even in asymptomatic patients [144]. Sadeghi et al. similarly showed that plasma levels of two tryptophan metabolites, kynurenine and quinolinic acid, correlated with the severity of CMV infection in kidney Tx recipients [150]. Moreover, patients experiencing pneumonia and/or sepsis showed significantly increased serum kynurenine levels already five days prior to diagnosis and treatment initiation compared to the conventional marker C-reactive protein (CRP), which increased only immediately before the start of therapy. Furthermore, the increase of kynurenine despite the ongoing antibiotics was associated with lethal endpoints [144]. Dharnidharka et al. also reported that the urine kynurenine/tryptophan ratio was significantly increased prior to major infection events in pediatric kidney transplant patients, even though the tryptophan metabolite levels in urine did not reflect acute rejection in this study [148]. Nevertheless, it is important to emphasize that kynurenine may not be suitable in the prediction or diagnosis of acute rejection or major infections in patients who are already dialyzed, because dialysis itself leads to elevated kynurenine levels [144,151].

Several attempts to use tryptophan and its metabolites as diagnostic or prognostic tools to predict graft function in a short-term or long-term period after Tx have been reported. A metabolomics study that enrolled [42] patients with or without acute kidney injury after renal Tx revealed the serum tryptophan and symmetric dimethylarginine levels to have the most significant negative correlations with blood urea nitrogen, serum creatinine and uric acid, being the most accurate markers for graft injury. When combined, these two markers reached an area under the curve of 0.901 with a sensitivity of 0.889 and a specificity of 0.831 for the diagnosis of acute kidney injury. These results implicate that decreased tryptophan levels may serve as a highly potential biomarker for graft injury [152]. Another group investigated the metabolomics of 40 human kidney allografts, looking for abnormalities in failing transplants. They found significantly reduced serum tryptophan levels in patients with low GFR after renal Tx. The decline in serum tryptophan was already detected in patients with slightly diminished GFR and became apparent in the group with the lowest GFR (21 ± 39 mL/min), revealing a dose response trend of tryptophan reduction. Similarly, a tryptophan decline in urine confirms that lower tryptophan levels reflect accelerated catabolism rather than urinary loss [11]. Vavrincova-Yaghi et al. focused on the prediction of chronic kidney allograft dysfunction and long-term kidney outcome. In general, the kynurenine/tryptophan ratio was significantly decreased at two years after Tx compared to two weeks and six months, thus reflecting the pattern of tryptophan metabolism changes. Serum and urine tryptophan and kynurenine levels, as well as the serum tryptophan/kynurenine ratio at two weeks and six months after renal Tx, were associated with two-year kidney graft function, as reflected by serum creatinine levels and albuminuria. Importantly, serum kynurenine at six months was found to be the only independent predicting factor for two-year serum creatinine. Interestingly, an association between the early levels of tryptophan or its metabolites with a histopathological level of graft damage was not found [145]. Another large prospective cohort study of 561 recipients with a more than one-year functioning renal graft revealed that baseline serum kynurenine, 3-HK and the 3-HK/kynurenine ratio were strongly associated with graft failure in long-term follow-up and kidney graft function. Out of all of these parameters, 3-HK was best-associated with long-term kidney graft outcomes. Serum 3-HK and the 3HK/kynurenine ratio were also linked to mortality, while serum kynurenine and the kynurenine/tryptophan ratio did not show such an association. However, the same parameters in urine did not show any such independent associations with long-term outcomes, suggesting that serum metabolites of the tryptophan-kynurenine pathway may be more favorable predictors in terms of kidney function [153].

Despite the fact that most of the predictive/diagnostic studies investigating tryptophan metabolism were conducted in the setting of kidney Tx, similar results were demonstrated in other transplant recipients. Meloni et al. investigated the plasma kynurenine/tryptophan levels in 26 patients with clinically stable lung grafts for >36 months after Tx and 64 patients who presented with bronchiolitis obliterans syndrome of various grades. A significantly increased kynurenine/tryptophan ratio was found in recipients with bronchiolitis obliterans syndrome in comparison to patients with stable grafts, supporting the idea that an increased IDO activity and tryptophan catabolism rather reflects chronic rejection than the protective/tolerogenic state of the immune system [154]. In a retrospective cohort study, Oweira et al. found significantly increased pre-Tx tryptophan, kynurenine, KYNA and quinolinic acid levels in patients who died due to sepsis or graft failure during the first year after liver Tx compared to those who survived more than one year. Together with serum bilirubin at post-Tx day five and phenylalanine at days five and 10, the pre-Tx serum kynurenine and kynurenic acid levels correlated significantly with one-year mortality after liver Tx [155].

In addition, tryptophan and its metabolites were also investigated as potential biomarkers of the organ quality and severity of graft IRI in livers. A metabolomics analysis of 37 human liver grafts revealed nearly two-fold increased levels of tryptophan and kynurenine in donation after circulatory death (DCD) grafts (in comparison with donation after brain-stem death (DBD) grafts) in the cold ischemia phase. All grafts were stored in tryptophan-free organ preservation solution, suggesting that tryptophan metabolism is probably responsible for the organ quality and may be used as a biomarker. Two primary nonfunctioning allografts in this cohort also had significantly higher levels of tryptophan and kynurenine in the cold phase. However, significant differences regarding tryptophan and its metabolites were not observed during reperfusion [10]. A metabolomics study investigating micro-dialysates of human liver grafts during the complex process of IRI revealed that kynurenine might potentially serve as a biomarker of aggravated ischemic injury. A nearly three-fold increased expression of kynurenine was found in DCD (donation after circulatory death) grafts at the end of cold ischemia compared to DBD (donation after brain-stem death) grafts. Moreover, the kynurenine levels gradually increased during reperfusion. Cold ischemia-phase kynurenine levels were also linked to graft outcome. Grafts that later developed a primary nonfunction or initially poor function had four-times higher kynurenine levels at 10 min of cold ischemia compared to immediately functioning ones [156]. There is an increased interest in the detection of biomarkers in the organ machine perfusion setting, which would allow determining the graft quality and predicting outcomes after implantation. As already mentioned before, a recent study found differences in tryptophan metabolism during discarded human liver normothermic machine perfusion compared to sub-normothermic machine perfusion. An increased tryptophan metabolism, as well as higher kynurenine and KYNA levels, have been found in livers subjected to normothermic compared to sub-normothermic machine perfusion. As grafts were not implanted, no associations with the later graft function were possible. However, this study suggests that measurements of tryptophan catabolites in perfusate could be used for graft quality assessment or the prediction of outcomes [24]. A recent experimental study using a mouse model raised concerns about the capability of tryptophan metabolism markers to reflect graft IRI. The authors found that metabolic profiles in acute cellular rejection and IRI after kidney Tx differ. Kynurenine was significantly increased during acute allograft rejection but not in IRI. This finding sheds some light on the potential discriminative property of tryptophan metabolites in distinguishing reasons for graft dysfunction [157].

The increased tryptophan metabolism seems to be paradox. On the one hand, it should work as an endogenous process inducing immune suppression and preventing graft rejection. On the other hand, studies have shown that increased kynurenine/tryptophan ratios and/or decreased tryptophan and/or increased levels of tryptophan metabolites reflect the graft rejection or injury. It raises a question if this IDO-dependent pathway is sufficient to achieve a tolerogenic state after solid organ Tx. Importantly, it should be taken into account that observational studies reveal correlations and associations between metabolite and graft functions or the event of rejection rather than causal relationships.

Most of the current evidence is available in the setting of kidney Tx, while sufficient data about the diagnostic role of tryptophan catabolism in other organ Tx settings is scarce. However, there is still a question whether tryptophan and its metabolites might be considered as markers in solid organ Tx, as the results of the studies are inconclusive. This may not only be explained by inconsistent types and sizes of study populations, endpoints of interest and varying follow-up periods but, also, by different measurement techniques. In general, the reliable and accurate quantification of tryptophan and kynurenine pathway metabolites in plasma, urine or organ perfusate might be challenging, especially in daily clinical practice, mainly due to their lability, low physiological levels and interference of other endogenous compounds in biological fluids [158]. Extreme concentration differences among tryptophan and its metabolites (e.g., plasma concentrations of tryptophan, kynurenine and 3HK in healthy individuals are about 60 µmol/L, 2 µmol/L and <0.13 µmol/L, respectively) [159] may hamper the simultaneous quantification of these analytes. Determination of the tryptophan-kynurenine pathway compounds mainly relies on chromatographic methods such as high-performance liquid chromatography (HPLC) coupled to ultraviolet (UV), fluorescent or electrochemical detection, as well as liquid or gas chromatography with mass spectrometric detection (LC-MS or GC-MS, respectively). However, specific drawbacks of all these techniques should be taken into account [155,158]. Although the high-performance LC with UV detection is easily accessible in clinical practice, its low sensitivity and specificity in tryptophan-kynurenine metabolite quantification due to the interference of other biological compounds, as well as the problems in the simultaneous determination of multiple metabolites, are major drawbacks of the method. Selectivity may be improved with fluorescence detection; however, not all kynurenines are fluorescent, making quantification without derivatization sometimes impossible. Electrochemical detection high sensitivity is an advantage for the detection of metabolites in low concentrations. However, its level of selectivity and reproducibility may be not satisfactory [158]. The most suitable and reliable chromatographic method for the simultaneous multi-compound analysis of tryptophan and kynurenine pathway metabolites is LC-MS, which allows the accurate quantification of analytes in a wider range of concentrations. However, due to the necessity of costly equipment, expensive isotope-labeled internal standards and precise sample preparation process, its utility in daily clinical practice is limited as well [11,146,147,149,152,158]. Nuclear magnetic resonance (NMR) is another promising technique that allows not only the rapid and precise quantification of various biochemical analytes in biological fluids or tissues but, also, the noninvasive in vivo analysis of metabolism in spectroscopic images and have also been successfully used to determine the levels of tryptophan-kynurenine pathway compounds [11,160]. Nevertheless, this method also requires sophisticated equipment, which currently limits its use mainly to the scientific setting.

Nevertheless, tryptophan metabolism via the kynurenine pathway remains an attractive potential indicator of graft quality and post-Tx function, as well as a reflector of the immune state. Therefore, we encourage the establishment of robust, accurate and universal detection techniques that could be available not only in scientific but, also, in clinical settings. Additionally, further studies validating tryptophan metabolites as noninvasive biomarkers in the field of Tx are needed.

**Table 3:** Tryptophan and its metabolite association with post-Tx outcomes in human studies.

Reference	Study Design	Population	Measurement	Follow-Up	Outcome Association			
					Graft Function	AR	Infection	Recipient Mortality
Holmes et al. [143], 1992	Retrospective	32 kidney Tx recipients	Post-Tx serum Trp and Kyn	3 weeks post-Tx	DGF: Trp↓, Kyn↑; SCr: Trp↓, Kyn↔	Trp↔, but ↓if failed AR treatment or concomitant infection; Kyn↑	CMV, EBV, pneumonia, UTI: Trp↓, Kyn↑	n.d.
Brandacher et al. [141], 2007	Prospective	43 kidney Tx recipients	Serum and urine Trp and Kyn 1, 8, 15 and 21 days post-Tx and at the time of AR	3 weeks post-Tx	n.d.	Serum Trp↓, Kyn↑, Kyn/Trp↓ Urine Kyn/Trp↓	H. simplex (n = 4), UTI (n = 1), sepsis (n = 1): serum Trp↔, Kyn↔, Kyn/Trp↔	n.d.
Lahdou et al. [140], 2010	Retrospective	210 first kidney Tx recipients	Pre-Tx plasma Trp, Kyn, post-Tx plasma Trp, Kyn, Kyn/Trp *available for 10 AR+ patients (median 7 days post-Tx) and 24 AR- patients (median 11 days post-Tx)	6 months post-Tx	n.d.	Pre-Tx Trp↑ (sens. 61%, spec. 71%), Kyn↑ (sens. 64%, spec. 71%); Post-Tx Trp↔, Kyn↑ (sens. 80%, spec. 79%), Kyn/Trp↑ (sens. 70%, spec. 79%)	n.d.	n.d.
Kaden et al. [144], 2015	Retrospective	355 kidney Tx recipients	Post-Tx serum Kyn	3 weeks post-Tx	DGF and PNF: slowed decrease or increase of serum Kyn	Kyn ↑ (level of increase strongly depends on AR severity in non-dialyzed patients)	CMV: Kyn↑ Pneumonia: Kyn↑ Sepsis: Kyn↑	n.d.
Vavrincova- Yaghi et al. [145], 2015	Prospective	48 kidney Tx recipients	Serum and urine Trp, Kyn, Kyn/Trp at 2 weeks, 6 months and 2 years post-Tx	24 months	SCr at 2 years: serum Kyn/Trp at 6 months↑; Kyn at 6 months↑ (AUC 0.76); CrCl at 2 years: urine Trp at 2 weeks↓ (AUC 0.44). Albuminuria at 2 years: Urine Trp↓; Kyn↑ at 2 weeks, Kyn/Trp↑ at 6 months	n.d.	n.d.	n.d.
Kim et al. [146], 2019	Cross-sectional	385 kidney Tx recipients	Urine metabolomic analysis (LC-MS)	n.d.	n.d.	TCMR: Trp↓; Panel of 5 top metabolites, including Trp showed AUC 0.926, acc. 87.0% (training set) and 62.5% (validation set)	n.d.	n.d.
Blydt-Hansen et al. [147], 2014	Cross-sectional	277 biopsy-paired urine samples from 57 pediatric kidney Tx recipients	Urine metabolomic analysis (LC-MS)	n.d.	n.d.	TCMR: Kyn↑; TMCR discriminant score of 10 top metabolites, including Kyn showed AUC 0.892	n.d.	n.d.
Dharnidharka et al. [148], 2013	Prospective	29 pediatric kidney Tx recipients	Serum and urine Trp, Kyn, Kyn/Trp	12 months after Tx	n.d.	AR within the next 30 days from the test: serum Kyn/Trp↑; urine Kyn/Trp↔; serum and urine Trp and Kyn↔	MIE (CMV, EBV, BKV, Tx pyelonephritis, fever with bacteriemia): serum and urine Kyn/Trp↔; serum and urine Trp and Kyn↔	n.d.
Zhao et al. [149], 2014	Cross-sectional	27 primary kidney Tx recipients	Serum metabolomics analysis (LC-MS) pre-Tx and 7 days post-Tx	n.d.	n.d.	In non-rejecting patients: Trp↓; Kyn↑; Kyn/Trp↑	n.d.	n.d.
Sadeghi et al. [150], 2012	Cross-sectional	86 kidney Tx recipients	Plasma Trp, Kyn, Quin, Kyn/Trp, Quin/Trp	n.d.	n.d.	n.d.	CMV: Trp↔; Kyn↑ (AUC 0.82), Kyn/Trp↑ (AUC 0.83) Quin↑ (AUC 0.85), Quin/Trp (sens. 83%, spec. 74%) (correlate with the severity); BKV: Trp, Kyn, Quin, Kyn/Trp, Quin/Trp↔	n.d.
Zhang et al. [152], 2018	Retrospective	42 kidney Tx recipients	Plasma metabolomics (25 amino acids) analysis (UHPLC–MS/MS)	n.d.	AKI (↑ of SCr of > 0.3 mg/dL or ↑ ≥50% over baseline): Trp↓ (AUC 0.78); Trp+SDMA↓ (AUC 0.901)	n.d.	n.d.	n.d.
Bassi et al. [11], 2017	Cross-sectional	40 kidney Tx recipients at least 6 months after Tx	Ex vivo (serum and urine) and in vivo metabolomics (LC-MS/MS, FIA-MS/MS (n = 40), 2D COSY with 3D-image transformation (n = 15)	n.d.	GFR 21–39 mL/min: serum Trp↓	n.d.	n.d.	n.d.
de Vries et al. [153], 2017	Prospective	561 stable kidney Tx recipients with functioning graft for at least 1 year	Serum and urine Trp, Kyn, 3-HK, Kyn/Trp, 3-HK/Kyn	Median 7.0 [6.2–7.5] years	Graft failure, SCr, proteinuria: serum Trp↓, Kyn, 3-HK, Kyn/Trp, 3-HK/Trp↑; no significant changes in urine; GFR: serum Trp↑, Kyn, 3-HK, Kyn/Trp, 3-HK/Trp↓	n.d.	n.d.	Serum Trp↔; Kyn; 3-HK; Kyn/Trp; 3-HK/Kyn↑; no significant changes in urine
Meloni et al. [154], 2009	Cross-sectional	90 lung Tx recipients	Plasma Trp, Kyn, Kyn/Trp	n.d.	n.d.	BOS: Trp↔, Kyn↑, Kyn/Trp↑	n.d.	n.d.
Oweira et al. [155], 2018	Retrospective	89 liver Tx recipients	Pre-Tx and early post-Tx serum Trp, Kyn, Quin, KYNA	1 year	EAD: pre-Tx Kyn↑ (AUC 0.64); post-Tx day 3 Kyn↑ (AUC 0.69); day 5 Kyn↑ (AUC 0.74); day 10 Kyn↑ (AUC 0.77)	n.d.	n.d.	1-year mortality: pre-Tx Kyn↑ (AUC 0.77), KYNA↑ (AUC 0.74), Quin↑ (AUC 0.72), Trp↑ (AUC 0.72); Post-Tx day 1 KYNA↑ (AUC 0.73); day 5 KYNA↑ (AUC 0.71); Kyn↑ (AUC 0.73). Pre-Tx Kyn↑ associated with 1-year mortality in univariate analysis
Perera et al. [156], 2014	Prospective	40 liver Tx recipients	Metabolomic analysis (CEAD) of liver micro-dialysis samples: pre-Tx (bench micro-dialysis) and every 6 h for 48 h post-Tx	7 days post Tx	PNF/IPF: pre-Tx (bench) Kyn↑; Kyn↑ in DCD vs. DBD grafts at the end of cold ischemia	n.d.	n.d.	n.d.

Abbreviations: ↑: positive association, ↓: negative association, ↔: no association, n.d.: no data, sens.: sensitivity, spec.: specificity, acc.: accuracy, AUC: area under the curve, Tx: transplantation, Trp: tryptophan, Kyn: kynurenine, Quin: quinolinic acid, KYNA: kynurenic acid, SDMA: symmetric dimethylarginine, LC-MS: liquid chromatography-tandem mass spectrometry, UHPLC–MS/MS: ultra-high performance liquid chromatography-tandem mass spectrometry, FIA-MS/MS: flow injection analysis-tandem mass spectrometry, 2D COSY: two-dimensional correlated spectroscopy, CEAD: coulometric electrochemical array detection, DGF: delayed graft function, PNF: primary nonfunction, EAD: early allograft dysfunction, IPF: initial poor function, SCr: serum creatinine, GFR: glomerular filtration rate, CrCl: creatinine clearance, AR: acute rejection, TCMR: T-cell-mediated rejection, BOS: bronchiolitis obliterans syndrome, MIE: major infection event, UTI: urinary tract infection, CMV: cytomegalovirus, EBV: Ebstein Barr virus, DCD: donation after circulatory death, DBD: donation after brain-stem death and BKV: BK virus.

## 6. Conclusions

It seems that the tryptophan metabolism via the kynurenine pathway may have multiple clinical effects in solid organ Tx, depending on the timepoint and the dominant pathophysiological mechanism (IRI or immune response against allograft), as well as the prevalent enzymes and metabolites of this metabolic pathway. However, this evidence grants some interesting insights into therapeutic possibilities by offering several attractive pharmacological and/or genetic modification targets, probably paving the way for protection from deleterious pathological effects in solid organ Tx and the improvement of short- and long-term outcomes. Moreover, metabolites of the tryptophan-kynurenine pathway may potentially serve as diagnostic and prognostic tools that allow improving and personalizing the care of transplanted patients. Further studies investigating this topic and answering the remaining questions are crucial.
